# Advancements of aquaporin 1 in ultrafiltration failure secondary to peritoneal dialysis

**DOI:** 10.1007/s00467-024-06626-9

**Published:** 2024-12-26

**Authors:** Tianxin Jiang, Jiahan Liu, Yuanyuan Shi, Lijie Zhang, Xinxin Xu, Jing Xiao

**Affiliations:** 1 Department of Nephrology, The First Affiliated Hospital of Zhengzhou University, Zhengzhou, Henan 450052 China; 2 The Renal Research Institution of Zhengzhou University, Zhengzhou, China

**Keywords:** AQP1, Ultrafiltration failure, Peritoneal dialysis, Water transport

## Abstract

For patients undergoing long-term peritoneal dialysis (PD), exposure to biologically incompatible PD solutions and the consequent peritoneal structure change can lead to progressive angiogenesis and fibrosis, and ultimately result in ultrafiltration failure (UFF). Peritoneal transport studies in aquaporin 1 (AQP1) knockout mice indicate that water transport across the peritoneum is mediated by AQP1, which accounts for up to 50% of ultrafiltration. Another recent study on a large cohort of PD patients with kidney failure further substantiated the impact of AQP1 genotype variation on water channel expression in the peritoneal membrane, influencing water transport, ultrafiltration, and patient prognosis. High-dose corticosteroid therapy in both patients and mice seems to be effective in regulating AQP1 to improve ultrafiltration. At present, increasing evidence suggests that AQP1 is relevant for the process of PD water osmotic transport and ultrafiltration. Despite a great deal of research having been done on the structure and function of aquaporin proteins, many fundamental issues remain unresolved.

**Graphical Abstract:**

**Figure:**
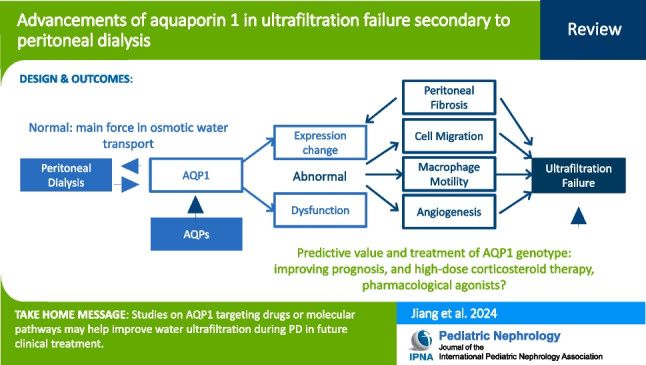
A higher resolution version of the Graphical abstract is available as Supplementary information

**Supplementary Information:**

The online version contains supplementary material available at 10.1007/s00467-024-06626-9.

## Peritoneal dialysis

### Epidemiology of peritoneal dialysis

As a globally recognized and increasingly serious public health issue, chronic kidney disease (CKD) affects 11 to 13% of the population worldwide [1]. With the advent of simplified techniques, such as the use of disposable plastic bags in continuous ambulatory peritoneal dialysis (CAPD), peritoneal dialysis (PD) has been recognized as a viable home-based kidney replacement therapy over the last 4–5 decades [2]. As of 2021, statistics indicate that patients undergoing PD account for approximately 11% of the global dialysis population [3].

The application of PD in children primarily focuses on transitional kidney replacement therapy before kidney transplantation. Due to its various dialysis modalities, suitability for home use, better preservation of residual kidney function, and advantages in facilitating a child patient’s travel and schooling, PD has become a powerful choice for dialysis treatment [4]. Interestingly, the peritoneal structure of children more closely adheres to the trajectory of pediatric growth and development, particularly evident in the total peritoneal microvessel density exhibiting a characteristic U-shaped curve. The density decreases initially and then increases with age, reaching its lowest point between the ages of 7 and 12. Nevertheless, the expression of mesothelial cell markers including aquaporin-1 (AQP-1) remains consistent across all age groups, indicating its distinct role in PD [5].

### Basic mechanisms of ultrafiltration and ultrafiltration failure

PD involves the exchange of solutes and water between the blood in the peritoneal capillaries and the dialysate within the peritoneal cavity, with the peritoneum serving as a semipermeable membrane. During this process, the ability to remove excess fluid via osmotic pressure is defined as ultrafiltration capacity. The three-pore model Rippe et al. established elucidates the transport mechanisms of water and solutes in PD [6]. Ultrasmall pores, a tetramer composed of AQP1, serve as water transport channels across the endothelial cell membrane, contributing to approximately 30 to 40% of total ultrafiltration, despite their limited number. These pores enable solute-free water transport via a transcellular pathway [7].

The peritoneum’s submesothelial capillaries define its effective surface area and serve as the primary, rate-limiting barrier for solute and water transfer [5]. During PD, solutes diffuse from the blood in the peritoneal capillaries into the dialysate, driven by an osmotic gradient created by glucose in the dialysate which also facilitates water removal from the blood. Over time, as glucose diffuses into the capillaries, the osmotic gradient diminishes and then reduces the ultrafiltration rate until equilibrium is reached. Thus, the density of peritoneal capillaries and the rate of glucose diffusion are critical for determining the ultrafiltration equilibrium and volume [8, 9]. Schaefer et al. found that despite the use of neutral pH low glucose degradation product (GDP) dialysate in children, a significant increase in capillary density occurred early during PD, and this vessel density correlated with peritoneal small-molecule transport function [9].

Under long-term PD, peritoneal capillaries gradually exhibit some vascular malformations, extracellular matrix expansion, as well as deposition of collagen fibers and advanced glycation end products in the vessel wall. These changes eventually lead to vascular lumen obliteration and significantly impair the peritoneal transport function [10, 11]. This phenomenon was observed and confirmed in peritoneal tissues of children exposed to high GDP dialysate [11]. Simultaneously, vascular lesions are also more likely to lead to progressive fibrotic changes, including loss of surface mesothelium and thickening of submesothelial dense areas, which may cause relative ischemia and further exacerbate fibrosis [10]. These structural changes in the peritoneum lead to a gradual decline in transport function, manifested by reduced solute clearance and ultrafiltration volume, ultimately resulting in ultrafiltration failure (UFF).

UFF is usually defined as less than 400 ml of net ultrafiltration after 4 h of 4.25% or 3.86% glucose PD fluid dwell in the abdomen for adults [12]. While in children, the definition changes to less than 150 ml of net ultrafiltration per m^2^ BSA during a standard 4-h peritoneal equilibration test (PET) using 2.3% glucose [13]. Adult studies suggest that about 38% of PD patients are diagnosed with UFF after 3 years of dialysis, and more than 50% of PD patients have manifestations of fluid overload to varying degrees [14, 15]. However, data on UFF in children are limited due to the generally shorter duration of PD before transplantation.

Four types of UFF have been described so far: (i) high effective peritoneal surface area, due to changes in peritoneal structure and increased capillary formation, results in rapid solute transport and reduced ultrafiltration capacity; (ii) low osmotic conductance to glucose, which is associated with impaired AQP1-mediated water transport and attenuation of sodium sieving; (iii) low effective peritoneal surface area, which features progressive fibrosis; (iv) high total peritoneal fluid loss rate, due to increased lymphatic and local tissue absorption, which is the most challenging to diagnose clinically [16].

With time on PD, accumulated uremic toxins [10], the high glucose environment of peritoneal dialysate [17], and advanced glycosylation end-products [18] can easily induce local inflammation. This inflammation promotes local neovascularization, leading to an increased and highly efficient peritoneal transport area, which results in rapid solute transport and the most common type 1 UFF. Rapid transport of small solutes is associated with collagen thickening and increased vascular permeability [12]. On the other hand, the non-physiological intraperitoneal environment also promotes vasculopathy, epithelial–mesenchymal transition (EMT) of mesothelial cells, and collagen deposition in the dense submesothelial zone, leading to fibrotic changes in the peritoneum and the development of type 3 UFF. Furthermore, a model of AQP1 knockout mice (AQP1^−/−^) shows that reduced peritoneal water permeability is associated with AQP1 dysfunction [19], a characteristic feature of type 2 UFF. And there is evidence suggesting a correlation between decreased AQP1 expression and the occurrence of neovascularization along with peritoneal fibrosis (PF) [20]. Consequently, patients may enter a state of UFF due to different factors mentioned above and may even coexist with multiple types of UFF [17]. Regardless of all types, UFF can lead to edema, chronic volume overload, and increased risk of congestive heart failure and cardiovascular mortality [21, 22].

## AQPs and AQP1

### Introduction to the AQPs

AQP, first identified in 1991, is a functional unit of water transport in the plasma membrane of human erythrocytes and kidney tubules [23, 24]. To date, 13 members of the water channel protein family have been identified in mammals with specific expression patterns and roles in particular tissues and cells. They are expressed in many epithelial and endothelial cells involved in fluid transport, as well as in cell types thought not to be involved in fluid transport, such as skin, fat, and bladder cells. Based on water and glycerol selectivity, the family of water channel proteins is commonly classified as “water channel proteins (AQP1, AQP2, AQP4, AQP5, AQP6, and AQP8)” and “water-glycerol channel proteins (AQP3, AQP7, and AQP9)” [24]. Water channel proteins and hydroglycerol channel proteins play a variety of roles in mammalian biology, including urine concentrating function [25], brain water homeostasis [26], cell migration [27], and glycerol transport in skin and fat [28, 29].

### Introduction to the AQP1

CHIP28 (AQP1), the first water channel protein to be discovered, is a water-specific channel protein [30]. It is a tetramer composed of four monomers, each containing six transmembrane α-helices and a pore which is selective for water, forming an hourglass-like structure arranged in a right-hand twist around the central pore. This hourglass-like structure is a key factor for its high permeability and strict selectivity for water [31]. Additionally, the tetrameric structure features a central pore that is reported to facilitate the transport of ions and small molecules such as potassium ion, oxygen gas, and ammonia gas [32, 33].

AQP1 is highly expressed in the epithelial cells of the proximal tubules and descending limb of Henle in the kidney. King et al. found impaired urinary concentration in two unrelated AQP1-deficient subjects [34], confirming AQP1’s essential role in urine concentration and water homeostasis. Additionally, AQP1 is also widely expressed in microvascular endothelial cells, including those in the kidney and tumor microvessels, as well as in non-vascular endothelial cells in the pleura, peritoneum [35], cornea, and lymphatic vessels. In recent years, it has been reported that AQP1 is also expressed in mesothelial cells, functioning as a water transporter [36–38]. Carlsson et al. demonstrated AQP1’s role in PD by inhibiting peritoneal water permeability in vitro using mercuric chloride in a rat model [39]. Recent studies have shown that AQP1 is involved in cell proliferation, migration [40, 41], invasion, wound healing [42], and angiogenesis [27].

## Regulation of AQP1 in PD water transport

### AQP1 plays an important role in osmotic water transport

Given AQP1’s crucial role in water transport during PD, its dysfunction in the peritoneal endothelium is a major cause of UFF [43]. Sieving of sodium, observed during PD, refers to the initial decrease in the dialysate/plasma (D/P) sodium ratio early in the PET with hypertonic dialysate containing 3.86% glucose. This phenomenon occurs at the peak of crystal osmotic pressure during the initial stage of PD, where AQP1-mediated water transport is most pronounced. Based on that, the extent of AQP1-mediated water transport can be estimated by the reduction in the D/P sodium concentration ratio [44]. A study using AQP1^−/−^ mice demonstrated that AQP1 mediates approximately 50% of ultrafiltration during the 2-h PET [19]. Zhang et al. used the Cre/loxP system to create an endothelium-specific AQP1 knockout mouse model and performed a 1-h mini-PET demonstrating that AQP1^−/−^ mice exhibited no sodium sieving and a 50% reduction in net ultrafiltration volume compared to WT mice [45]. All these confirm the significance of AQP1 in sodium sieving and ultrafiltration during PD.

### AQP1 in PD failure

As previously mentioned, AQP1 is involved in 50% of the water transport during ultrafiltration. Nevertheless, studies have shown no significant difference in AQP1 expression between healthy controls and PD patients [35, 46]. Goffin et al. also found no significant difference in the expression of AQP1 of a 67-year-old man with UFF compared with control patients without ultrafiltration problems except for its higher glycosylated expression [47]. In another recent study conducted on mice with diabetes and hypercholesterolemia, it was observed that the expression level of glycosylated AQP1 was significantly higher. This increase in glycosylated AQP1 was regulated by glucose hyperosmotic stress and then promoted the development of atherosclerosis in the mouse aortas [48]. In contrast, high glucose-stimulated mesothelial cells (HUVECs) have been shown to downregulate AQP1 [49]; an increase in the expression of AQP1 in mesothelial and endothelial cells (EA.hy926) after stimulation with high glucose has also been demonstrated [50]. Despite extensive research on water channel proteins, many fundamental questions remain unanswered. For instance, the specific expression trend of AQP1 in UFF and the impact of modifications on AQP1 dysfunction still need to be determined.

## Complications of PD and AQP1

### AQP1 modulates cell migration

Peritonitis is a common complication of PD and is associated with increased small solute transport due to microvascular proliferation, leading to a larger effective peritoneal surface area, faster glucose reabsorption, and early resolution of the osmotic gradient, ultimately reducing ultrafiltration. Saadoun et al. observed reduced angiogenesis in AQP1^−/−^ mice in subcutaneous and intracranial tumor models. While adhesion and proliferation were similar in primary cultures of aortic endothelial cells from wild-type and AQP1^−/−^ mice, cell migration was significantly impaired in AQP1-deficient cells, with abnormal angiogenesis in vitro [27]. The molecular pathways by which AQP1 influences peritoneal endothelial cell migration remain unclear but may involve specific membrane expression patterns.

In addition to endothelial cells, evidence of water channel protein-dependent migration has been found in epithelial cells of the kidney proximal tubule [40] and astrocytes [41]. Despite water channel proteins, such as AQP1 and AQP4, being involved in cell migration by facilitating rapid changes in cell volume, the normal development of AQP1 and AQP4 null mice suggests they are not essential for this process. Additionally, retinal angiogenesis in a neonatal hyperoxia model is not dependent on AQP1, further confirming that AQP1 is not required for cell migration [51].

EMT is a key mechanism in the development of PF [52] characterized by the loss of epithelial-specific markers and the acquisition of a fibroblast phenotype with increased cell motility [52, 53]. However, the role of AQP1 in regulating cell migration in EMT remains to be confirmed.

### AQP1 regulates macrophage motility

Macrophages play a crucial role in immunity by migrating to and phagocytosing at sites of infection or injury, and they can switch between two main phenotypes: (i) classically activated macrophages (M1), which are pro-inflammatory, anti-tumor and anti-microbial, and (ii) alternatively activated macrophages (M2), which are anti-inflammatory and involved in tissue remodeling and repair. Tyteca et al. found that AQP1 deletion in peritoneal macrophages from AQP1^−/−^ mice affected their morphology and migration, with a twofold reduction in infiltrating macrophages compared to WT mice [42]. These results suggest that AQP1 is required for macrophage functions critical for tissue remodeling and wound healing.

### AQP1 promotes angiogenesis

In long-term PD patients, the peritoneum undergoes structural changes including increased collagen fiber density, diameter in the mesangial layer, and peritoneal vasculature proliferation. These changes increase the effective peritoneal transport area, a major risk factor for rapid solute transport. Saadoun et al. found that AQP1^−/−^ mice implanted with melanoma cells exhibited slower tumor growth and improved survival compared to WT mice [27]; with reduced microvessel density in tumor tissue, speculating that AQP1 deficiency impairs tumor angiogenesis and leads to extensive necrosis. However, in AQP1^−/−^ mice treated with PET, vascular density was not significantly altered [19]. Therefore, the role of AQP1 in promoting angiogenesis, particularly in the peritoneum, requires further investigation.

## Potential therapeutic strategies for AQP1-regulated water transport

### PD prognosis predicted by AQP1 genotype

The efficiency of PD depends on the peritoneal membrane’s ultrafiltration capacity, which removes excess water. However, there is significant variability in water and solute transport during early PD, affecting dialysis regimens and prognosis. Morelle et al. collected clinical and genetic information from 1851 patients across 7 cohorts and found that the rs2075574 mutation in *AQP1* was associated with peritoneal ultrafiltration capacity. Patients with the TT genotype had a mean daily net ultrafiltration 200 ml lower than those with the CC genotype. Despite inter-cohort heterogeneity, the predictive value of *AQP1* genotypes for PD outcomes was justified. This suggests that common *AQP1* promoter variants may influence water channel expression in the peritoneum, affecting water transport, ultrafiltration, and prognosis in PD-treated kidney failure patients [54].

### Adjusting AQP1 to improve ultrafiltration

Given the critical role of AQP1 in UFF, regulating AQP1 to enhance ultrafiltration is significant. Stoenoiu et al. found that the *AQP1* gene promoter contains a glucocorticoid-responsive element, and corticosteroids induce AQP1 expression in the peritoneal membrane, improving water permeability and ultrafiltration in male Wistar rats. Additionally, dexamethasone treatment for 5 days in rats increased their capillary endothelial AQP1 expression, resulting in significantly enhanced water transport and net transmembrane ultrafiltration [55].

Researchers have also studied the water and solute transport in PD patients treated with high-dose glucocorticoids, focusing on patients before and after kidney transplantation. They found that sodium sieving doubled in 3 patients undergoing above treatment after kidney transplantation compared to that before transplantation, along with an increase in AQP1-mediated ultrafiltration volume, while the rate of small solute transport remained unchanged [56].

Although progress on AQP1 pharmacological agonists has been slow, AqF026, a furosemide derivative, has been identified as the first effective agonist. In the Xenopus oocyte system, extracellular application of 5 to 20 mM of AqF026 increased human AQP1 activity by over 20%. In a mouse PD model, AqF026 enhanced osmotic water transport without affecting the osmotic gradient, small solute transport, or AQP1 expression and localization. The lack of potentiation in AQP1-null mice suggests that AqF026 specifically targets AQP1 [57]. This finding may offer new therapeutic insights for PD and related water transport issues.

In summary, both high-dose glucocorticoid therapy and AQP1 pharmacological agonists offer new perspectives for future clinical improvements in water transport and peritoneal ultrafiltration. However, these findings require further validation in larger patient cohorts, and the systemic effects of high-dose steroids must be considered. Future research should focus on investigating AQP1 regulators, their specific mechanisms, and potential effects on different cell types.

## Conclusions and prospects

AQP1 mediates up to 50% of ultrafiltration during PD, making its expression and function clinically significant. However, recent studies have not clearly determined the specific trend in AQP1 expression in the peritoneum. Rodent models, with their thinner peritoneum and faster transport rates, may not accurately represent human UFF, and thus, drawing conclusive findings is complicated. Further clinical investigations are needed to confirm AQP1 expression changes in UFF.

**Fig. 1:**
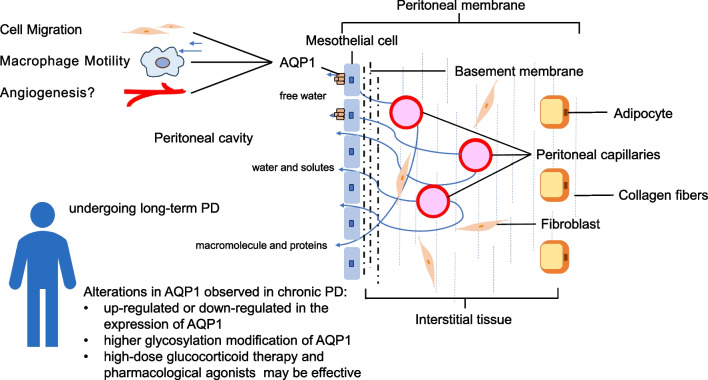
A summary of the role of peritoneal AQP1 from a physiological perspective and the alterations in AQP1 observed in chronic PD

For another, in the high-glucose microenvironment of long-term PD, advanced glycosylation end products or other mechanisms may alter AQP1 structure, potentially affecting its osmotic water transport function and explaining the loss of sodium sieving. Additionally, AQP1’s roles in cell migration, wound healing, tumor growth and dissemination [27] may contribute to EMT; vascular proliferation, and leukocyte recruitment during inflammation [42], in conjunction with peritonitis, can lead to UFF (Fig. 1).

Currently, the role of AQP1 in pediatric PD is underexplored, despite PD being a common transitional therapy before kidney transplantation. Given the predictive value of *AQP1* genotypes in PD outcomes [54], future research should focus on *AQP1* genotyping in children with stage 5 CKD to assist in more appropriate kidney replacement therapy strategies.

In conclusion, studies on AQP1-targeting drugs and molecular pathways may benefit future clinical treatment of PD. Specifically, research should focus on how aberrant glycosylation of AQP1 and its interaction with junctional proteins as well as signaling pathways [33] might regulate osmotic water transport by altering AQP1’s molecular structure.

## Supplementary Information

Below is the link to the electronic supplementary material.

Graphical abstract (PPTX 75 KB)

## Data Availability

The data that support the findings of this study are available from the corresponding author upon reasonable request.
