# Supplementary material for: Advancements of aquaporin 1 in ultrafiltration failure secondary to peritoneal dialysis
Source: Pediatr Nephrol. 2024 Dec 26;40(6):1863–9. doi: 10.1007/s00467-024-06626-9 (PMC12031960; doi:10.1007/s00467-024-06626-9)
Supplement: Supplementary file 1 — Graphical abstract (PPTX 75 KB) [file 467_2024_6626_MOESM1_ESM.pptx]

## Slide 1
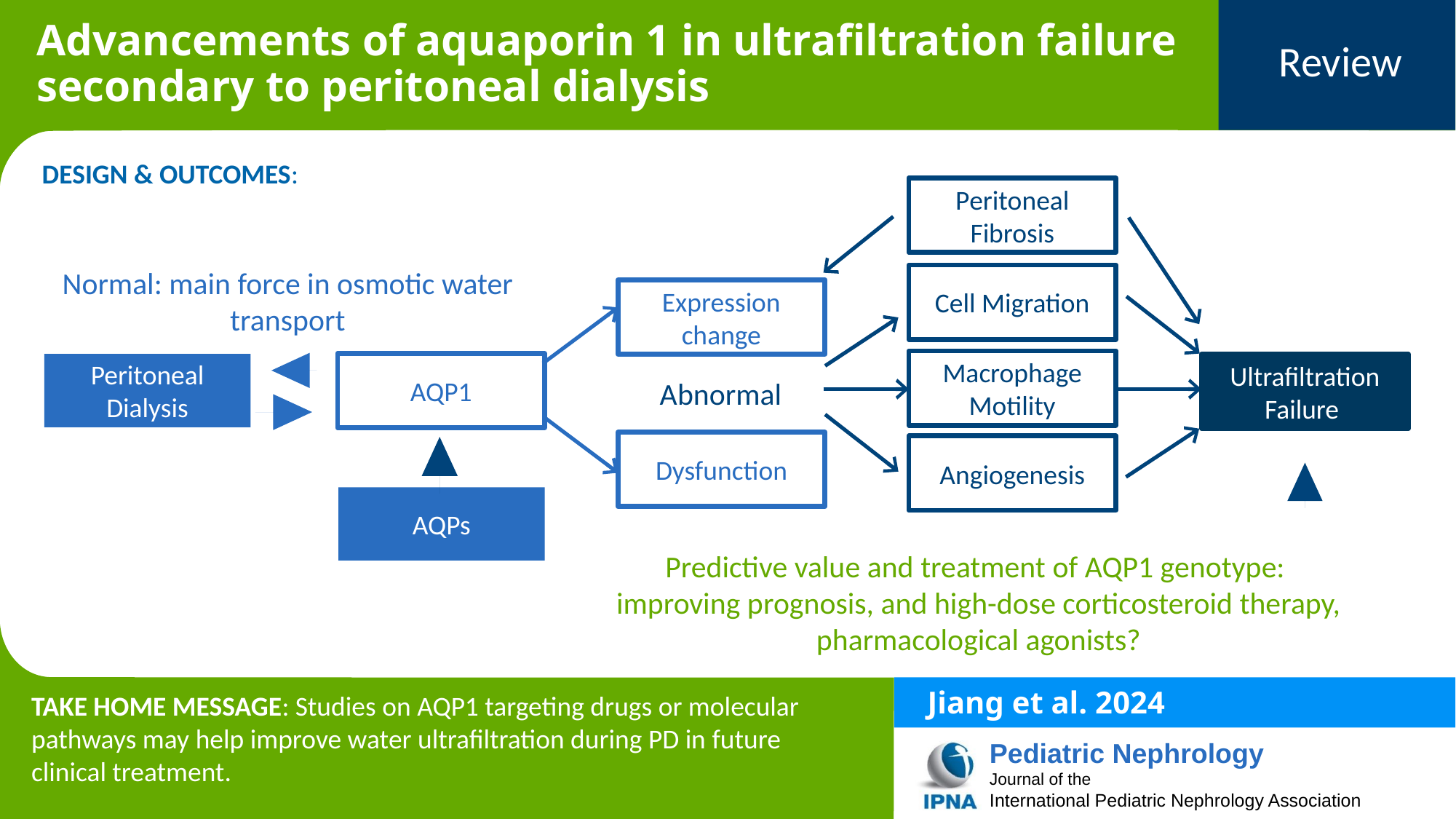

Advancements of aquaporin 1 in ultrafiltration failure secondary to peritoneal dialysis
DESIGN & OUTCOMES:
Peritoneal Fibrosis
Normal: main force in osmotic water transport
Cell Migration
Expression change
Macrophage Motility
AQP1
Peritoneal Dialysis
Ultrafiltration Failure
Abnormal
Dysfunction
Angiogenesis
AQPs
Predictive value and treatment of AQP1 genotype:
improving prognosis, and high-dose corticosteroid therapy, pharmacological agonists?
Jiang et al. 2024
TAKE HOME MESSAGE: Studies on AQP1 targeting drugs or molecular pathways may help improve water ultrafiltration during PD in future clinical treatment.
